# Neurological update: treatment escalation in multiple sclerosis patients refractory to fingolimod—potentials and risks of subsequent highly active agents

**DOI:** 10.1007/s00415-021-10956-1

**Published:** 2022-01-09

**Authors:** Melanie Korsen, Steffen Pfeuffer, Leoni Rolfes, Sven G. Meuth, Hans-Peter Hartung

**Affiliations:** 1 Department of Neurology, Medical Faculty, Heinrich-Heine-University Düsseldorf, Moorenstraße 5, 40225 Düsseldorf, Germany; 2 LWL-Clinics Muenster, Muenster, Germany; 3 Brain and Mind Centre, University of Sydney, Sydney, Australia; 4 Department of Neurology, Medical University of Vienna, Vienna, Austria; 5 Department of Neurology, Palacky University Olomouc, Olomouc, Czech Republic

**Keywords:** Adverse events, Disease-modifying treatment, Effectiveness, Fingolimod, Multiple sclerosis, Switch

## Abstract

A critical issue in the management of relapsing MS (RMS) is the discontinuation of disease-modifying treatments (DMT) due to lack of efficacy, intolerability or impending risks. With new therapeutic agents introduced into the treatment of RMS, immediate- and long-term consequences of sequential drug use, as well as the effect of the sequence in which the drugs are given, are unclear but may affect efficacy, adverse events, and long-term immunocompetence. In the absence of clinical studies specifically addressing these concerns, observations from clinical practice are of particular value in guiding current management algorithms. Prompted by a study published by Ferraro et al. in this journal, we set out to provide an overview of the published real-world evidence on the effectiveness and safety of switching from fingolimod to another DMT in patients with active RMS. Seventeen publications reporting relevant information were identified. The literature suggests that immune cell depletion induced by alemtuzumab or ocrelizumab is associated with an increased risk of relapse and worsening disability in patients switching from fingolimod compared to patients switching from other therapeutic agents. However, the evidence reported for natalizumab and cladribine is inconclusive. While shortening of the washout period may limit early disease reactivation after fingolimod discontinuation, there is no strong evidence that the duration of the washout period or the absolute lymphocyte count at baseline are predictors of attenuated long-term efficacy. Further real-world studies are required to better understand outcomes among patients who are under-represented in controlled trials.

## Introduction

The expansion of the treatment landscape in active relapsing multiple sclerosis (RMS) has led to increased complexity of treatment choices. Treatment options currently compromise immune cell sequestering substances, such as the sphingosine-1-phosphate receptor blocker fingolimod and the monoclonal antibody natalizumab, as well as cell-depleting agents, such as the small molecule drug cladribine and the monoclonal antibodies alemtuzumab (anti-CD52), ocrelizumab (anti-CD20) and ofatumumab (anti-CD20) [1]. They can be administered as a first-line treatment, but most patients are initially treated with low-risk platform agents and then switched to these drugs due to insufficient disease control (so-called ‘treatment escalation’). While several studies have examined this treatment strategy [2, 3]—the progression from first- to second-line therapy—evidence regarding a ‘lateral switch’ to a drug with comparable efficacy but different mode of action, following previous active treatment, remains scarce. This is mainly due to phase III clinical trials supporting the approval of these disease-modifying therapies (DMT) not usually including patients on prior active treatment. Consequently, considerations regarding efficacy as well as a comprehensive risk–benefit assessment critically depend on the evaluation of data collected by comparative observational studies done in the real-world setting [4, 5]. A ‘lateral switch’ from fingolimod to another DMT may be necessary due to different reasons including lack of efficacy, family planning or safety issues. As guidelines do not make recommendation on this matter and clear data regarding treatment sequencing are missing, we consider it of utmost importance to give a compact overview of current data to help physicians in their daily routine when advising patients on fingolimod regarding a necessary ‘lateral switch’.

Two general considerations have to be taken into account regarding efficacy when switching from one highly active DMT to another. First, the transition period between cessation of the existing DMT and initiation of a new agent must balance the efficacy, safety and the risk of recrudescence of disease activity or rebound. For instance, many real-world studies have reported disease reactivation after the discontinuation of fingolimod [6]. A second point for consideration is whether a previous drug could nullify or attenuate the mode of action of a subsequent therapy. Here, the question arises whether immune cell-depletion may be less effective if initiated immediately after the use of fingolimod as both the sequestration-dependent effects and those independent of sequestration are overlapping.

Beyond efficacy concerns, real-world data are also required to assess the potential impact of switching from one DMT to another on patient safety. As most active DMTs have lasting or even irreversible effects on patients’ immune systems [7, 8], the cumulative impact of subsequent monotherapies with different mechanisms of action makes it difficult to predict the clinical consequences of highly active DMTs administered in succession.

Given the increasing complexity of treatment options and the fact that clear guidance to manage the switch from the second-line sequestering treatment agent fingolimod to other compounds is lacking, we here focus on the role of fingolimod pre-treatment on the efficacy and safety profile of the subsequent highly active agents used in RMS. We further comment on management considerations, such as baseline lymphocyte count and washout duration, to assist and optimize the decision-making process in clinical practice. Real-world and observational studies are herein reviewed to understand outcomes among patients who are potentially under-represented in controlled trials.

## Methods

We conducted a MEDLINE search to identify relevant articles published between January 1, 2011 and August 31, 2021. The Medical Subject Headings (MeSH) terms applied were ‘fingolimod’ and ‘switch’ or ‘escalation’ or ‘sequence’ or ‘natalizumab’ or ‘cladribine’ or ‘alemtuzumab’ or ‘ocrelizumab’ or ‘ofatumumab’. Additionally, we decided to include eligible studies sourced from international conferences (the Annual Meeting—American Academy of Neurology (AAN) and the European/Americas committee for treatment and research in multiple sclerosis (ECTRIMS/ACTRIMS)), personal communications with the authors, and consultation of national and international registries for clinical trials [United States National Library of Medicine (NLM); clinicaltrials.gov; European Union Drug Regulating Authorities Clinical Trials Database (EudraCT)].

Articles that were included met the following criteria: (i) adult patients with active RMS according to 2010 or 2017 revised McDonald criteria [9, 10]; (ii) previous treatment with fingolimod according to national and international guidelines as well as the summary of product characteristic (SmPC); (iii) a switch to natalizumab, cladribine, alemtuzumab, ocrelizumab and ofatumumab.

**Fig. 1:**
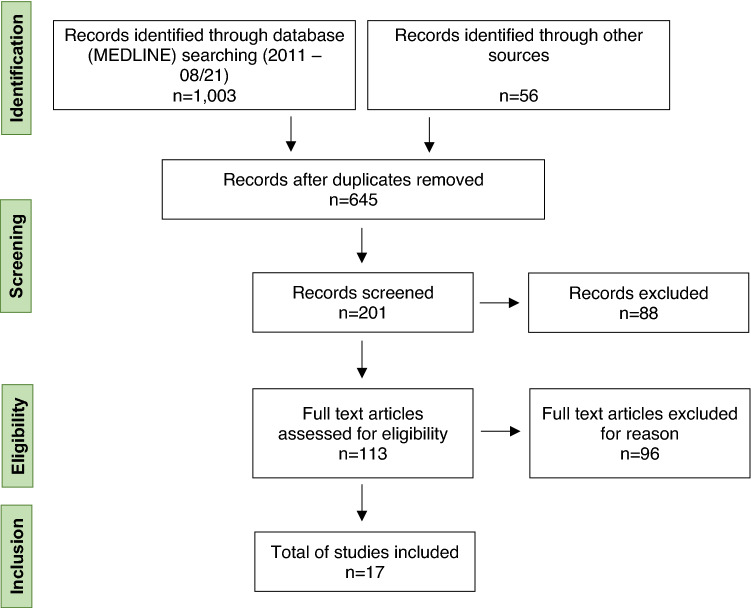
PRISMA flow diagram of the reviewed literature

Our literature search returned 1,059 records in total. Among these studies, 17 records met our inclusion criteria (for details on the search strategy see Fig. 1).

### Effectiveness and safety in patients switching from fingolimod to other DMT approved for active RMS

Efficacy of fingolimod has been proven in various clinical trials [11–13]. However, in the real-world setting, up to 50% of patients show ongoing disease activity within the first year of treatment [14, 15] and probably require a switch to another DMT. Furthermore, reasons like safety issues or family planning might make it necessary to switch DMT. Vollmer et al*.* did a 36-month follow-up on 535 fingolimod patients and observed that ~ 21% of all patients discontinued fingolimod due to intolerance, ~ 10% due to other reasons which might likely include family planning among others [16]. Wicks et al*.* performed an online survey in an online patient community [17]. 62 patients currently on fingolimod and 32 patient which discontinued fingolimod participated. 46.9% of the latter discontinued fingolimod treatment due to side effect, 25% due to of lack of effectiveness, 6.3% due to the advice of their doctor and 15.5% due to other reasons including family planning.

#### Switch to natalizumab

While switching to fingolimod after discontinuation of natalizumab has been reported in a number of studies, there are currently no real-world data published on switching from fingolimod to natalizumab compared to treatment-naïve patients being given natalizumab (Table 1). Similarly, the pivotal clinical trials of natalizumab, AFFIRM [18] and SENTINEL [19] only enrolled treatment-naïve patients or those treated with beta-interferons or glatiramer acetate. Thus, at present, no valid assessment can be made as to whether natalizumab is a favorable option to choose from the approved monoclonal antibodies when considering patients who are on previous active therapy.

**Table 1:** Overview of publications on fingolimod treatment sequences in active relapsing multiple sclerosis

Drug and author et al	Study title	Study design	Subjects, Total No. (No. FTY pre-treatment)	Primary outcome	FTY as risk factor for disease reoccurrence		Impact of washout duration	Lymphopenia as risk factor for disease reoccurrence	Safety concerns	Follow- up duration	References
					During wash-out	During treatment					
Natalizumab	–										
Cladribine Pfeuffer et al.	Effectiveness and safety of cladribine in MS: Real-world experience from two tertiary centers	Prospective multicenter	270 (18 FTY)	Time to CWD, relapse, MRI activity* and loss of NEDA3^a^, compared to naïve patients	N/A	No	N/A	No	No	42 months	[22]
Cellerino et al.	Severe disease activity in MS patients treated with cladribine after fingolimod withdrawal	Retrospective case series	3 (3 FTY)	EDSS progression, Relapse rate	Yes	Yes	N/A	N/A	N/A	6 months	[24]
Radlberger et al.	Immune phenotyping study revealing caveats regarding a switch from fingolimod to cladribine	Case report	1 (1 FTY)	EDSS progression, Relapse rate, lymphocyte (subtype) counts	Yes	Yes	N/A	N/A	N/A	12 months	[25]
Alemtuzumab Willis et al.	An observational study of alemtuzumab following fingolimod for multiple sclerosis	Retrospective multicenter	36 RMS (36 FTY)	Relapse rate, MRI activity*	N/A	Yes	N/A	No	N/A	12 months	[28]
Bernard-Valnet et al.	Unexpected high multiple sclerosis activity after switching from fingolimod to alemtuzumab	Case report	1 RMS (1 FTY)	N/A	Yes	Yes	N/A	N/A	N/A	6 months	[29]
Wehrum et al.	Activation of disease during therapy with alemtuzumab in 3 patients with multiple sclerosis	Retrospective case series	3 RMS (2 FTY)	EDSS progression, Relapse rate, MRI activity*	N/A	Yes	N/A	N/A	N/A	12 months	[30]
Eichau et al.	Efficacy of Alemtuzumab in patients who switched from Fingolimod	Retrospective monocenter	50 RMS (25 FTY)	Relapse rate, EDSS progression compared to other compounds	No	No	N/A	No	N/A	12 months	[31]
Alemtuzumab Huhn et al.	Alemtuzumab as rescue therapy in a cohort of 50 relapsing–remitting MS patients with breakthrough disease on fingolimod: a multi-center observational study	Retrospective multicenter	50 RMS (50 FTY)	Percentage of patients with relapses or MRI activity compared to the previous year	No	No	N/A	No	No	12 months	[32]
Frau et al.	Outcomes after fingolimod to alemtuzumab treatment shift in relapsing–remitting MS patients: a multicentre cohort study	Retrospective multicenter	77 RMS (77 FTY)	Percentage of patients with relapses, EDSS progression or MRI activity* compared to the previous year	N/A	No	No	No	N/A	12 months	[33]
Alcalá et al.	Treatment with alemtuzumab or rituximab after fingolimod withdrawal in relapsing–remitting multiple sclerosis is effective and safe	Retrospective multicenter	55 RMS (55 FTY, switch to Alemtuzumab in 28)	Percentage of patients with relapses, CWD, MRI activity* and NEDA3^a^ loss, compared to patients switching to rituximab	N/A	No	No	No	No	24 months	[34]
Pato et al.	Efficacy and safety of alemtuzumab after switching from a second-line therapy	Prospective multicenter	59 RMS (16 FTY)	Percentage of patients with relapses or MRI activity compared to patients switching from natalizumab	N/A	No	N/A	N/A	N/A	12 months	[35]
Theodorsdottir et al.	Alemtuzumab treatment in Denmark: a national study based on the Danish MS Registry	Prospective multicenter (Danish MS Registry)	210 RMS (95 FTY)	Proportion of patients that needed a 3^rd^ cycle	N/A	Yes	N/A	N/A	N/A	42 months	[36]
Alemtuzumab Pfeuffer et al.	Impact of previous disease-modifying treatment on effectiveness and safety outcomes, among patients with multiple sclerosis treated with alemtuzumab	Prospective multicenter	170 RMS (33 FTY)	Time to CWD and relapse, compared to naïve patients	N/A	Yes	No	No	Yes	42 months	[37]
Ocrelizumab Signoriello et al.	Switch from sequestering to anti-CD20 depleting treatment: disease activity outcomes during wash-out and in the first 6 months of ocrelizumab therapy	Retrospective multicenter	165 RMS (110 FTY)	Relapse rate, EDSS progression, MRI activity*	Yes	Trend toward higher relapse rates	Yes	Yes	N/A	6 months	[38]
Boudot et al.	Challenges of switching toward anti-CD20 monoclonal antibodies in RR-MS: A monocentric study	Retrospective monocenter	73 RMS (31 FTY)	Percentage of patients with relapses	Yes	N/A	Yes	N/A	N/A	No FU	[39]
Schmidt et al.	Severe rebound after cessation of fingolimod treated with ocrelizumab with coincidental transient aggravation: report of two cases	Retrospective case series	2 RMS (2 FTY)	Relapses, EDSS progression, MRI activity*	Yes	Yes	N/A	N/A	N/A	3 months	[40]
Various Ferraro et al.	Risk of Multiple Sclerosis relapses when switching from fingolimod to cell-depleting agents: the role of washout duration	retrospective	329 RMS (329 FTY)	Time to first relapse	No	No	Yes	N/A	N/A	22 months	[49]

*CWD* confirmed worsening of disability, *EDSS* Expanded Disability Status Scale, *FTY* fingolimod, *FU* Follow-up, *MRI* magnetic resonance imaging, *N/A* not applicable, *NEDA3* no evidence of disease activity, *No* number, *RMS* relapsing–remitting multiple sclerosis

*MRI activity was either defined as gadolinium-enhancing lesions and/or new or enlarging T2 lesions

^a^No evidence of disease activity as defined by Havrdova et al. [23]

#### Switch to cladribine

Cladribine is a synthetic purine analog prodrug approved for the treatment of active RMS since 2017. However, the supporting randomized clinical trials were initiated in 2010 and did not include any of the newer DMTs [20, 21]. Regarding the switch from fingolimod to cladribine, a prospective evaluation of 270 RMS patients recently demonstrated a good safety profile and effectiveness [22]. Outcome parameters were: time to confirmed worsening of disability, first relapse, paraclinical activity or loss of NEDA (no evidence of disease activity)-3 status [23] compared to treatment-naïve patients or those previously treated with injectables. To ensure that disease activity was not predominantly driven by rebound following cessation of the last previous immunotherapy, re-baselining to month six was performed. Following fingolimod pre-treatment, patients experienced mainly paraclinical disease activity after the treatment switch; however, disease stability occurred for most patients after having passed month six. While early re-occurrence of disease activity during the washout period was confirmed by several case reports during the switch to cladribine [24, 25], the absence of disease reactivation after the initiation of treatment contrasted the results of Pfeuffer et al. [22]. Regarding safety considerations, both fingolimod and cladribine likely exert their clinical efficacy by depleting peripheral immune cells. However, fingolimod pre-treatment was neither a risk factor for the development of severe lymphopenia nor for the occurrence of herpes infection upon cladribine initiation [22].

#### Switch to alemtuzumab

Alemtuzumab was shown to be highly efficacious in controlling disease activity among both treatment-naïve patients (CARE-MS I) and those who had a poor response to a first-line DMT (CARE-MS II) [26, 27]. Patients enrolled in the CARE-MS II trial had been previously treated mainly with beta-interferon or glatiramer acetate; none of them received fingolimod [26].

In the real-world setting, a British observational study by Willis et al*.* first raised concerns that the escalation to alemtuzumab might not achieve good control of disease activity [28]. The authors showed that 9 out of 36 patients experienced significant disease activity within the first twelve months after switching from fingolimod to alemtuzumab. While the mean relapse rate significantly increased during the first year of treatment, the second dose of alemtuzumab subsequently reduced the rate below pre-treatment values. In line with this, several case reports [29, 30] described significant and unexpected disease activity following switching from fingolimod, mainly around month six after alemtuzumab induction [29, 30]. Subsequently, several studies were conducted to assess the efficacy of alemtuzumab in patients previously treated with fingolimod, with conflicting results. While four retrospective analyses [31–34] and one small prospective study [35] demonstrated good effectiveness of alemtuzumab following fingolimod, a Danish prospective registry study showed that patients previously exposed to fingolimod are more likely to require a third course of alemtuzumab due to ongoing disease activity compared to other compounds [36].

Notably, none of the aforementioned studies performed comparisons with previously naïve or platform-treated patients. In this context, we have recently published a large, prospective cohort study demonstrating that patients previously treated with fingolimod had a worse response to alemtuzumab compared with other treatment groups (including naïve patients and those who had received injectables), as they experienced a less marked reduction in relapse rate and higher hazard ratios for worsening disability [37]. Additionally, we observed an ‘altered relapse pattern’, as patients previously exposed to fingolimod were more likely to develop spinal relapses, contributing to their increased hazard radio for worsening disability.

Regarding the safety profile, and considering the contrasting results from three retrospective analyses mentioned above [32–34], we found that patients who had received fingolimod were prone to developing secondary autoimmunity, with no specific patterns of autoimmunity between treatment groups [37].

#### Switch to anti-CD20 antibodies

Regarding B cell depletion by anti-CD20 antibodies, an observational multicentre study recently assessed disease activity in 165 individuals treated with ocrelizumab following discontinuation of fingolimod or natalizumab [38]. The authors demonstrated that previous treatment with fingolimod increased relapses during washout and also noted a tendency toward increased relapse occurrence during the first 6 months of ocrelizumab therapy compared to patients switching from natalizumab. A similar trend of increased relapse rate during washout when switching from fingolimod to a B cell-depleting therapy compared to other compounds was further reported by Boudot et al. [39]. In this retrospective monocentre study, 20 patients (27.4% of the total patients included) with different previous treatments experienced relapses during the washout period, with a probability of relapse post-fingolimod of 35% 1 month after withdrawal.

In support of this, Schmidt et al*.* reported two cases of breakthrough disease during treatment with fingolimod who then experienced a severe rebound after fingolimod withdrawal with further significant clinical deterioration after ocrelizumab initiation [40].

It should be noted that a study has neither reported a favorable outcome when switching from fingolimod to ocrelizumab nor studied switching to ofatumumab—the safety profile of these treatment sequences is yet to be analyzed. Results from controlled clinical trials of ocrelizumab and ofatumumab are also unhelpful, as only 5 out of 825 patients in OPERA I and II [41] and 23 out of 946 patients in ASCLEPIOS I and II [42] were pre-exposed to fingolimod.

### Possible mechanisms of compromised efficacy and resulting management considerations

Since a substantial proportion of lymphocyte subsets is sequestered in secondary lymphoid tissues by fingolimod, ongoing lymphopenia by sphingosine-1 phosphate receptor blockade probably results in reduced accessibility of cells for depletion and might therefore be a potential risk factor for suboptimal treatment response. Hu et al*.* showed that lymphocyte depletion by alemtuzumab was markedly less profound in lymphoid organs than in peripheral blood using a transgenic mouse model expressing human CD52 [43]. Regarding anti-CD20-agents experimental models have shown that there is likely a more direct access to lymph nodes with subcutaneous administration than with intravenous infusion [44, 45].

In total, seven of the included studies conducted a subgroup analysis regarding the absolute baseline lymphocyte count of patients with or without evidence of persistent clinical or MRI disease activity during subsequent treatment [22, 28, 32–34, 37, 38]. Indeed, Signoriello et al*.* showed that lymphocyte counts at baseline influence early treatment effectiveness of ocrelizumab [38]. However, in all other studies, lymphocyte counts at baseline did not differ significantly [22, 28, 32–34, 37]. Accordingly, in most of the studies indicating reduced effectiveness of either alemtuzumab [28, 29, 37] or ocrelizumab [38, 40], lymphocyte counts were already at the lower limit of normal at the beginning of treatment. However, the total blood lymphocyte count does not depict differences in lymphocyte subsets and their respective tissue distribution. Yet, none of the included studies performed detailed immunophenotyping to address this issue. Only one case report described that re-emerging disease activity after cladribine was associated with accelerated repopulation of naïve B cells and lower pre-treatment T cell and memory B cell levels [25]. Similarly, recurrent disease activity under alemtuzumab treatment was associated with high B cell counts in three other patients [30].

Another widely debated aspect is the impact of the washout period on disease reactivation upon fingolimod discontinuation [46]. On the basis of a reversible immune reconstitution for fingolimod [47], and the risk of disease reactivation when washout is prolonged [48], a short period between two treatments could be considered to minimize rebound activity. Indeed, shortening the time period when switching to anti-CD20 therapies after fingolimod reduced relapses between the cessation and initiation of the new treatment [38, 39]. Of note, this relationship was not investigated for other treatment switches.

On the other hand, and in line with aspects presented above, a longer washout period could lead to recovery from lymphopenia before initiation of cell-depleting agents, which may limit the risk of therapies being ineffective. Interestingly, a recent study by Ferraro et al. [49] in this journal showed that extending the washout period prior to commencing cell-depleting agents does not positively affect outcome parameters in the long term. In this study, the risk of relapse increased with the washout duration when switching from fingolimod to a lymphocyte-depleting agent (including cladribine, alemtuzumab, rituximab and ocrelizumab) during the 22 months of follow-up. In contrast, disease activity during alemtuzumab therapy did not correlate with the washout interval in other studies [33, 34, 37].

## Discussion

Since prospective, randomized, controlled clinical trials are not available, this review summarized current real-world evidence regarding the best treatment switch strategy following fingolimod.

This current review highlights the dilemma that patients on fingolimod in need of a switch toward another DMT have few therapeutic options. It was shown that patients switching from fingolimod to ocrelizumab were more likely to experience suboptimal disease control and worsening of disability throughout the washout period and during a follow-up period of 6 month [38–40]. Use of alemtuzumab as successor to fingolimod was repeatedly debated [28–36]. However, a large prospective cohort study confirmed impaired effectiveness and safety of this therapeutic switch [37]. In contrast, cladribine remained effective and safe following fingolimod treatment [22], but—although no study yet examined the cumulative risk of adverse skin events—cladribine predisposes to dermatological side effects, including skin tumors [50, 51], and thus concerns remain. Finally, natalizumab may remain an option but there is no real-world evidence yet that this switch is beneficial. Regarding feasibility and safety issues here, it should be considered that natalizumab carries a risk of progressive multifocal leukoencephalopathy (PML). Among fingolimod patients included in recent studies, up to 25% previously received and stopped natalizumab due to John Cunningham Virus (JCV) seropositivity and subsequent safety concerns [52, 53]. Cases of PML occurred in patients receiving fingolimod in the post-marketing setting who had neither been previously treated with natalizumab nor were taking immunosuppressive or immunomodulatory medications concomitantly [54]. It is currently unknown whether or how the sequencing of these therapies might impact the overall PML risk for each patient. Data examining the outcome after a switch form fingolimod to another DMT in regards to the reason for the switch either being treatment inefficiency or safety concerns and personal matters such as family planning are missing. A differentiation between these patient groups appears crucial to the further understanding of an individualized treatment strategy.

Interestingly, one study analyzed the effect of re-dosing on disease activity [28] and demonstrated a reduced risk of relapse after the second dose of alemtuzumab. One explanation may be that after a period of time, sequestrated lymphocytes eventually become available for targeting. However, the contribution of lymphocyte sequestration by fingolimod to suboptimal disease control following cell-depleting treatment remains questionable and our literature search suggests this view is too simplistic. Further, no correlation between baseline CD19^+^ B cell levels and fingolimod pre-treatment in relapsing or stable patients were found upon ocrelizumab initiation [38].

However, it is likely that the cellular immune status (as performed in routine clinical practice) is not sufficient to determine the underlying mechanisms of disease re-occurrence. For example, in the past, a more detailed assessment of B cells in the periphery including CD19^+^ CD27^+^ memory B cells was shown to be more reliable in prediction of clinical activity [55]. In this context, potential similar memory B cell depletion mechanisms were further described with cladribine and alemtuzumab [56]. Moreover, previous data indicate that fingolimod also exerts several effects on B cells, including induction of a regulatory phenotype [57], reduction in memory B cells [58] and increased production of anti-inflammatory cytokines [59]. Therefore, during disease activity, it may be plausible that the removal of regulatory B cells from the peripheral immune system by the subsequent therapy contributes to relapses at the beginning of treatment. Conversely, pre-treatment with natalizumab was shown to not only increase peripheral B cell numbers but also induce a more pro-inflammatory phenotype of B cells [60]; patients undergoing the switch from natalizumab to ocrelizumab experienced sufficient disease control [38]. Other qualitative changes in the immune network that may influence disease activity include transcriptomic changes of CD4^+^ T cells [61], the modulation of T helper cell phenotype balances and the increase in regulatory T cell abundance [62]. We speculate that these effects interfere with cell depletion and immune reconstitution in an unfavorable manner, resulting in increased risk for progression of disability [24, 25, 28–30, 36–40] and, perhaps in part, the development of secondary autoimmunity [37].

## Conclusion and outlook

There are numerous conclusions to be drawn from this review. First, the optimal successive treatment for patients with the need of a therapeutic switch while receiving fingolimod still needs to be defined. Evidence for natalizumab is lacking and, as it carries the risk of PML development, it may not be a longer-term alternative. Alemtuzumab also seems to represent a suboptimal option. Further studies evaluating whether ocrelizumab remains impaired in its effectiveness beyond re-treatment in month six and whether cladribine post-fingolimod is associated with a poor safety profile are necessary. Future studies should furthermore distinguish between the different reasons for treatment discontinuation. Efficacy after switching from fingolimod to another DMT may differ depending on the reason being disease activity or safety issues or family planning while the disease is basically stable.

Second, shortening of the washout period when switching from fingolimod may reduce relapse occurrence between cessation and initiation of the new treatment; however, regarding longer-term efficacy, data are contradictory. Therefore, further studies regarding the effect of blood lymphocyte subsets and their respective tissue distribution on the efficacy of the follow-up treatment after fingolimod are needed. 

Finally, prescribing DMTs in RMS depends on a thorough risk–benefit analysis, which is inconclusive if the patient’s characteristics are not reflective of clinical trial populations. Therefore, the question is raised whether patients with RMS previously treated with DMTs, other than what are considered platform agents, should be enrolled in clinical trials more regularly. Synthetic comparator arms for control groups could help to maintain feasibility here. Furthermore, it appears that DMTs more likely to require an ‘ongoing evaluation’ in the real-world setting, as new DMTs are continuously being introduced and consequently concerns regarding treatment switches cannot be clarified by extension studies alone. Here, implementation of international registries would add value to the existing evidence and improve data quality as the discussed data mainly originate from single center studies with smaller patient numbers. Such approaches would also be more likely to result in the recognition of safe succession of therapies. To date, data regarding ‘safe successions’ in a way represent negative reporting and it follows that they despite the best of intentions have been published less frequently.

In light of missing national and international guideline recommendations and taking all available real-world data into account, some centers prefer the following pathway: closely monitored washout period including immunmonitoring, lymphocyte counts above 800/µl, if possible above 1000/µl at initiation of new DMT, switch to B cell-depleting DMT after washout period.

As the treatment landscape for RMS evolves quickly, the question of recommendations on ‘lateral switches’ on patients under highly effective DMTs will become particularly important during the future. Therefore, high-quality data on efficacy and safety of ‘lateral switches’ and a joint concept how to collect these kinds of data internationally to obtain large patient numbers are needed.

## Data Availability

As stated in the method section, data were obtained from MEDLINE, international conferences, personal communications with the authors, and consultation of national and international registries for clinical trials.
